# Crosslinking strategies for preparation of extracellular matrix-derived cardiovascular scaffolds

**DOI:** 10.1093/rb/rbu009

**Published:** 2014-10-20

**Authors:** Bing Ma, Xiaoya Wang, Chengtie Wu, Jiang Chang

**Affiliations:** State Key Laboratory of High Performance Ceramics and Superfine Microstructure, Shanghai Institute of Ceramics, Chinese Academy of Sciences, 1295 Dingxi Road, Shanghai 200050, People’s Republic of China

**Keywords:** extracellular matrix, tissue engineering, scaffold, cross-linking agent, calcification

## Abstract

Heart valve and blood vessel replacement using artificial prostheses is an effective strategy for the treatment of cardiovascular disease at terminal stage. Natural extracellular matrix (ECM)-derived materials (decellularized allogeneic or xenogenic tissues) have received extensive attention as the cardiovascular scaffold. However, the bioprosthetic grafts usually far less durable and undergo calcification and progressive structural deterioration. Glutaraldehyde (GA) is a commonly used crosslinking agent for improving biocompatibility and durability of the natural scaffold materials. However, the nature ECM and GA-crosslinked materials may result in calcification and eventually lead to the transplant failure. Therefore, studies have been conducted to explore new crosslinking agents. In this review, we mainly focused on research progress of ECM-derived cardiovascular scaffolds and their crosslinking strategies.

## Introduction

Cardiovascular disease such as heart valve disease, coronary heart disease and heart failure is the main cause of morbidity and mortality. Current valve substitutes for replacing the diseased heart valves mainly include mechanical prostheses and bioprosthetic valves, such as allograft valves and xenograft valves [1, 2]. Mechanical valves are associated with significant risk of thromboembolic complications and need lifelong anticoagulation therapy [3], and they also lack the ability to grow, repair and remodel, which limits their long-term application in human body [4–6]. The reduced availability of allografts due to donor scarcity remains significant challenge for cardiovascular disease treatment. Both the porcine aortic valves and bovine pericardial tissues as a part of xenograft valves do not require the treatment of anticoagulation, which could enhance survival and quality of life patients, especially the pediatric patients with congenital [2, 7]. However, these valves are usually far less durable and frequently undergo calcification associated with progressive structural deterioration [2, 8]. The blood vessels used for transplantation mainly come from the patient’s vein, internal mammary and radial artery, etc [9]. But the available autologous blood vessels from patients themselves are always limited. In order to solve the problem of insufficient source of autologous vein grafts, synthetic grafts and xenogenic tubular tissues are widely used in constructing tissue engineering blood vessel. The synthetic materials included polyethylene terephthalate (Dacron®), expanded polytetrafluoroethylene and polyurethane [10]. These materials are successfully applied for large-diameter arteries replacement (>6 mm), and they possess the advantages of good biocompatibility, easy formation of desired shapes and ready availability. However, these synthetic materials are not degradable, and not suitable for small-diameter blood vessel replacement (<6 mm) due to thrombogenicity [11]. Other synthetic materials, which are widely used for constructing porous scaffolds, are biodegradable polymers, including polylactic acid (PLA), polyglycolic acid (PGA), polycaprolactone, polyhydroxyalkanoates and polyhydroxybutyrate [12]. PGA and PLA are commonly used in cardiovascular tissue engineering because their degradation products are metabolized and eliminated easily. However, PGA tends to lose its mechanical strength within 4 weeks, and needs long time to be completely absorbed.

The xenogenic valve and tubular tissues such as porcine heart valves, bovine pericardium and carotid arteries, have the advantage of being readily available. However, the raw tissues may induce immunogenicity after implantation *in vivo*, so decellularized materials, which mainly are extracellular matrix (ECM), are often used as cardiovascular scaffolds [13–15]. The decellularization could remove the cellular components and retain the structure of ECM, but it also lead to the loss of ECM integrity and degenerative structural failure, and the ECM-derived scaffolds may be calcified after implantation [7, 13]. In order to avoid these limitations, ECM-derived scaffolds are crosslinked using crosslinking agents to prevent degeneration, enhance mechanical strength and reduce calcification [16]. In recent years, significant progress has been make in the development of ECM-derived scaffolds for cardiovascular tissue replacement and tissue engineering, especially in the development of new crosslinking strategies. Therefore, the aim of this review is to discuss the development of crosslinking strategies for preparation of ECM-derived cardiovascular scaffolds.

## Crosslinking Strategies

An ideal biomaterial crosslinking agent should be no cytotoxicity and have low cost. It could improve the mechanical performance of the materials and inhibit calcification. There are many crosslinking agents for fixing ECM-derived scaffolds, which may be classified as (i) chemical crosslinking agents and (ii) natural crosslinking agents. The chemical crosslinking agents include glutaraldehyde (GA), carbodiimide (1-ethyl-3-(3-dimethyl aminopropyl)-carbodiimide (EDC)), epoxy compounds, six methylene diisocyanate, glycerin and alginate, etc. [17–21]; and the natural crosslinking agents include genipin (GP), nordihydroguaiaretic acid (NDGA), tannic acid and procyanidins (PC).

### Chemical crosslinking agents

#### Glutaraldehyde

**Figure 1.**
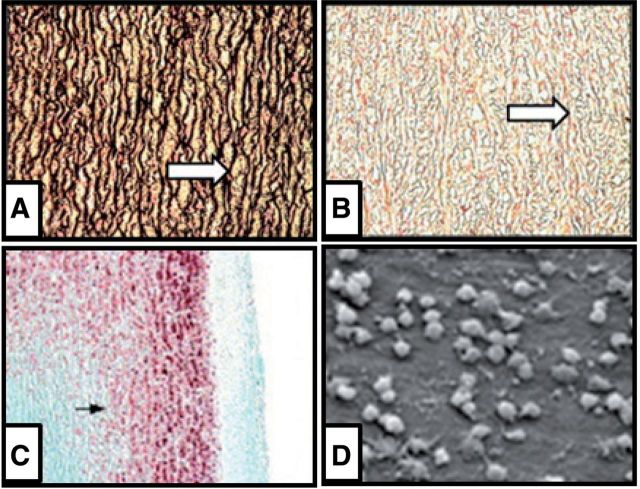
Disadavantage of GA-crosslinked ECM. Histology of GA-treated aortic samples before (**A**) and after (**B**) elastase. Calcium deposition in subdermally implanted GA-treated aorta explanted at 21 days (**C**). Scanning electron microscopy of human endothelial cells on GA-treated bovine pericardium shows sporadic cell cadavers (**D**). (Reprinted with permission from Refs. [22, 50].)

GA has been extensively used as a crosslinking agent to fix bioprosthetic valves and bovine pericardium, and it can significantly improve mechanical strength and durability of the ECM-derived scaffolds [22, 23]. GA reacts with amino groups available in protein molecules, which helps to form a more tightly crosslinked network between many protein molecules [23]. The ECM-derived scaffolds fixed with GA could significantly improve tensile strength and pliability and reduce antigenicity [22]. GA crosslinking can make scaffolds non-resorbable and non-amenable for its ability to resist to matrix metalloproteinase, but it cannot resist to elastase (Fig. 1A and B). In addition, the toxicity of GA (Fig. 1C) and GA-induced calcification (Fig. 1D) limits the long-term implantation, and results in final failure of the implant [23].

Detoxifying strategies have been proposed to increase the biocompatibility and durability of the GA-fixed ECM-derived scaffolds, and many methods were used to improve cellular adhesion and proliferation on GA-fixed scaffolds, including pretreatment with citric acid or amino acid solutions to remove free aldehyde groups [24–26]. Although these methods aim to develop a detoxifying treatment for GA-fixed ECM-derived scaffolds and have positive effects on the improvement of biocompatibility, they do not have obvious effect on calcification of GA-fixed materials [26].

The mechanism of calcification of GA-fixed ECM-derived scaffolds is complex. It is considered that there are many factors such as phospholipids, free aldehyde groups, and residual antigenicity play important roles in inducing calcification [27]. There are many methods for inhibition of calcification of GA-crosslinked scaffolds. Since the phospholipids and cholesterol are two substances which are thought to induce calcification, ethanol or its solutions have been used to extract these molecules from the tissue to inhibit the calcification [28, 29]. Some studies have shown that elastin may play a key role in tissue calcification and some calcification-related diseases such as atherosclerosis and heart valves calcification have been found associated with elastin degradation [30]. Therefore, trivalent metal ions (Fe^3+^ or Al^3+^) are used, in which stable crosslinking forms between Al^3+^/Fe^3+^ and elastic proteins in order to stabilize the microstructure of elastic protein [30].

#### 1-Ethyl-3-(3-dimethyl aminopropyl)-carbodiimide

EDC is a kind of compounds that contain a double bond which can react with many groups, such as carboxyl, hydroxyl and sulfydryl groups [31]. EDC fixation of ECM-derived scaffolds mainly involves the activation of carboxyl groups of glutamic and aspartic acid residues in the peptide chain and formation of *O*-acylisourea intermediate which can be nucleophilic attacked by free amino groups of lysine or hydroxyl lysine residues [32]. In addition, EDC crosslinking forms a network crosslinking which effectively increases the mechanical stability of collagen material and prevents the movement of macromolecules and infiltration of water molecules [20]. *N*-hydroxysuccinimide, an affinity reagent, added to EDC solution will effectively increase the number of crosslinks [21]. EDC-crosslinked ECM-derived scaffolds are soft and similar to natural tissue materials, and reveal low residual toxicity, which is in favor of the recellularization of scaffolds [23, 32]. Olde Damink *et al.* [21] have shown that the EDC-crosslinked collagen can resist enzymatic degradation of collagen matrix *in vitro*. However, EDC crosslinking may not be able to inhibit calcification, which limits its potential application in preparation of cardiovascular grafts [19].

#### Epoxy compounds

Epoxy compounds have multiple epoxy functional groups which can react with amino, carboxyl and hydroxyl groups. Epoxy compounds have been used to preserve biological tissue materials and this new fixation technique has recently been employed to crosslink biological heart valves and vascular grafts [17, 18]. Epoxy compounds crosslinked ECM-derived scaffolds are white, soft and no shrink, in which the collagen maintains loose and natural state [33]. However, epoxy compounds crosslinking is linear and has low crosslinking degree, and it cannot resist enzymatic degradation of collagen and has poor stability [33]. In addition, epoxy compounds in crosslinked ECM-derived scaffolds have shown certain toxicity and will cause immune response and calcification, which is similar to GA [18, 23, 34].

### Natural crosslinking agents

Natural substances as crosslinking agents show superiority in many aspects, especially in terms of cytotoxicity and anti-calcification ability. Many different kind of natural crosslinking agents such as GP, NDGA, tannic acid and PC have been studied for crosslinking cardiovascular scaffolds.

#### Genipin

GP is obtained from the natural compound geniposide which extracted from the fruit of Gardenia jasminoides Ellis, and it is one of the active ingredients of the traditional Chinese medicine extraction. It belongs to the iridoid compounds, which have multiple active groups, such as hydroxyl and carboxyl [35]. The applications of GP crosslinking acellular ECM in tissue engineering have been reported [36]. GP can spontaneously react with the free amino groups of lysine, hydroxyl lysine and arginine within some biomaterials and generate iridoid nitrides, and then form intramolecularly and intermolecularly crosslinking by the polymerization process with a heterocyclic structure [36, 37]. GP prefers to form annular crosslinking, which is more stable than the reticular crosslinking formed by GA and the linear crosslinking formed by pEPC. GP-crosslinked ECM-derived scaffolds, which are dark blue pigments, have ability of resistance to enzymatic degradation *in vitro*. Compared with GA, GP-crosslinked ECM-derived scaffolds have lower inflammatory response, and would not release the GP in the process of preservation [36, 38, 39]. However, the dark blue appearance of GP-crosslinked ECM-derived scaffolds, together with the exorbitant price and the complex extraction process, will greatly limit its application for treatment of cardiovascular tissues.

#### Nordihydroguaiaretic acid

NDGA, which is isolated from the creosote bush, is natural plant polyphenol compounds containing two functional ortho-catechols at the ends of a short alkane [40–42]. It has antioxidative and anticancer activity, and has the ability to crosslink collagen fibers, which results in an increase of the mechanical properties of the tissue matrix [42]. NDGA crosslinks collagen fibers by forming bisquinone crosslinking between NDGA molecules first, which further form a crosslinked NDGA network, and collagen fibers are then firmly embedded into this network to form a stable matrix [43]. The crosslinked collagen fibers appear brown, which is similar to natural collagen fibers. NDGA crosslinking concentration will directly affect the mechanical tensile strength and hardness of collagen [40]. However, NDGA at the concentration above 100 µM was cytotoxic to cells [41].

#### Tannin acid

Tannin acid (TA) is d-glucose gallic acid ester containing multiple phenolic hydroxyl groups and aromatic rings. It is widely found in fruits, seeds of leguminous plant, grain and a variety of drinks (such as wine, tea, cocoa and apple juice) [44, 45]. TA has a relatively high molecular weight, and can interact with carbohydrate, proteins and other biological macromolecules [44]. The term ‘tannin’ was first proposed in 1796. It was originally known because it can react with collagen protein and transform the animal’s skin into leather, which is therefore the original leather tanning method [46]. The mechanism of interaction between TA and collagen is mainly through hydrogen bonding and hydrophobic effects, which is influenced by the molecular weight and three-dimensional space structure [47, 48]. TA also has obvious crosslinking effect on elastin and improves its stability and enzyme degradation resistance [49]. TA can significantly reduce the calcification of GA-crosslinked aortic elastin after *in vivo* transplantation [50].

#### Procyanidins

Flavonoids are plant secondary metabolites widely distributed in fruits and vegetables [51]. Recently, the researchers realized that flavonoids can crosslink the cardiovascular scaffold materials, which could solve the disadvantages of GA, such as residual toxicity and calcification. PC, one of the most important flavonoids, is widely researched currently [52–54].

PC is an oligomer or polymer composed of flavan-3-ol (e.g. epicatechin or catechin), and it is naturally occurring plant metabolites widely available in fruits, vegetables, nuts, seeds, flowers and barks. The main characteristic of PC is that it can produce anthocyanin after heating in the acid medium, so it is named PC [45]. Most foods contain exclusively B-type PC which is linked by C4–C8 and/or C4–C6 bonds, and a small number of foods contain A-type PC which contain an additional ether bond between C2 and O7. Polymeric forms of PC are the predominant existence in many foods that contain PC [55]. High-pressure liquid chromatography analyses demonstrated that grape seed PC extract contains approximately 75–80% oligomeric PC and 3–5% monomeric PC [56].

**Figure 2.**
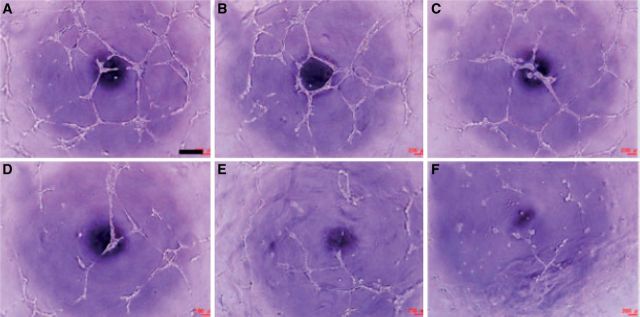
*In vitro* angiogenesis in the presence of PC at 0 (**A**), 0.1 (**B**), 0.5 (**C**), 1.0 (**D**), 1.5 (**E**) and 100 (**F**) µg/ml. Scale bar is 400 µm. (Reprinted with permission from Ref. [59].)

**Figure 3.**
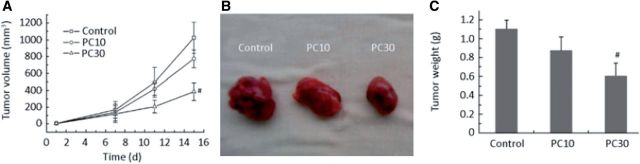
Anti-tumor effect of PC on tumor volume (**A**), tumor morphology (**B**) and tumor weight (**C**). PC 10 and PC 30 are 10 and 30 mg PC/kg bodyweight, respectively. Number sign indicates *P* < 0.05 compared with control. (Reprinted with permission from Ref. [59].)

PC displays a variety of biological activities, such as antioxidant, anti-inflammatory, anti-bacterial, anti-tumor, anti-calcification and cardiovascular protection effects. PC can reduce the expression and secretion of MMP-2 which have been demonstrated as an angiogenic factor. In addition, PC can also inhibit the activity of MMP-2 [57]. In addition, many studies have shown that PC could effectively inhibit tumor angiogenesis (Fig. 2), and angiogenesis-mediated tumor growth (Fig. 3) [58, 59]. Teissedre *et al.* [60] reported that PC extracted from grapes and red wine have remarkable scavenging activities and could inhibit oxidative modification of LDL *in vitro*. Therefore, dietary containing PC is important for maintaining health and reducing the incidence of atherosclerosis by their antioxidant activity, and decrease the risk of cardiovascular disease [56, 61].

## PC Crosslinking of ECM-Derived Scaffolds

### Stability and biocompatibility of PC-crosslinked scaffolds

**Figure 4.**
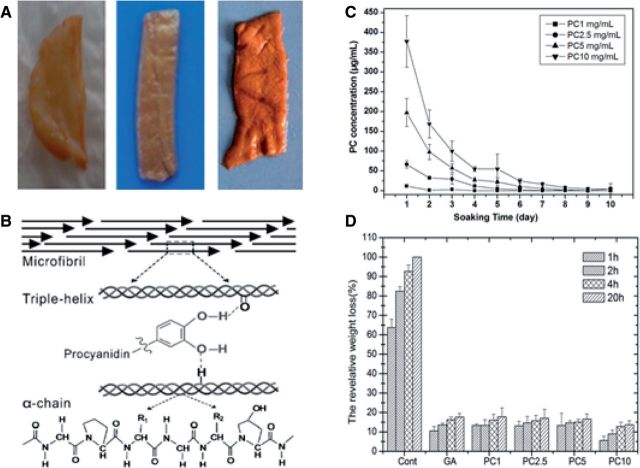
PC-crosslinked decellularized tissue scaffolds and the crosslinking mechanism and their stability. (Reprinted with permission from Ma Bing *et al.* Journal of East China Normal University 2013;**5**:61–79. Ref. [62]. L. He *et al.* International Journal of Biological Macromolecules 2011;**48**:354–359. W.Y. Zhai *et al.* Journal of Biomedical Materials Research Part B: Applied Biomaterials 2014;**102**:1190–8.)

PC-crosslinked decellularized cardiovascular scaffold materials (Fig. 4A), including decellularized porcine aortic heart valves, bovine pericardiums and blood vessels have been successfully prepared. PC-crosslinked ECM-derived scaffolds show palm red, soft and stretch, and do not shrink [52, 62]. Han *et al.* [63] have reported that PC could crosslink collagen (Fig. 4B) and no calcification appears in PC-crosslinked collagen after implanting in rat subcutis. Through the treatment of detergent or hydrogen bonding weaken agent, the interaction between PC and collagen could be damaged, which suggests that PC crosslinking mechanism might be related to the hydrogen bonding interaction which is mainly formed between protein amide carbonyl and PC phenolic hydroxyl groups. Proline molecule, which is a good hydrogen bond receptor, has a carbonyl oxygen adjacent to the amino groups. Collagen is rich in proline, so it can form stable hydrogen bonds with PC [64].

**Figure 5.**
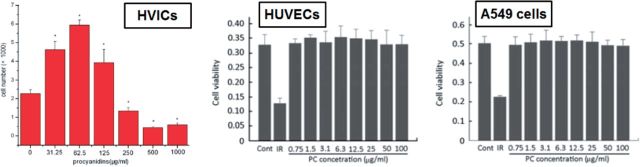
The proliferation effect of PC on bovine aortic heart valve interstitial cells (HVICs), HUVECs and A549 cells. IR is 1.5 µg/ml irinotecan. (Reprinted with permission from Refs. [59, 62].)

**Figure 6.**
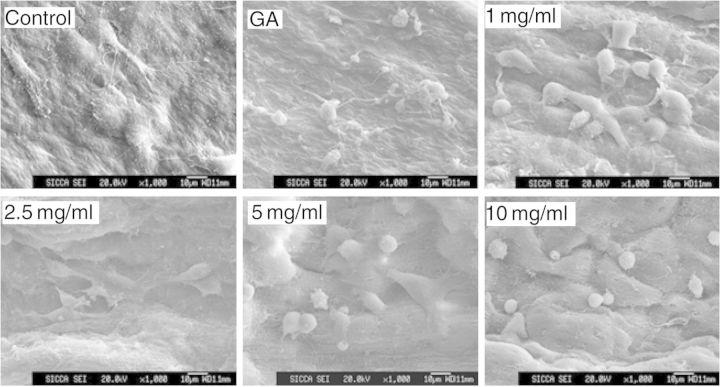
SEM images of the biocompatibility of PC-crosslinked decellularized arotic scaffold materials seeding with HUVECs. (Reprinted with permission from W.Y. Zhai *et al.* Journal of Biomedical Materials Research Part B: Applied Biomaterials 2014;**102**:1190–8.)

**Figure 7.**
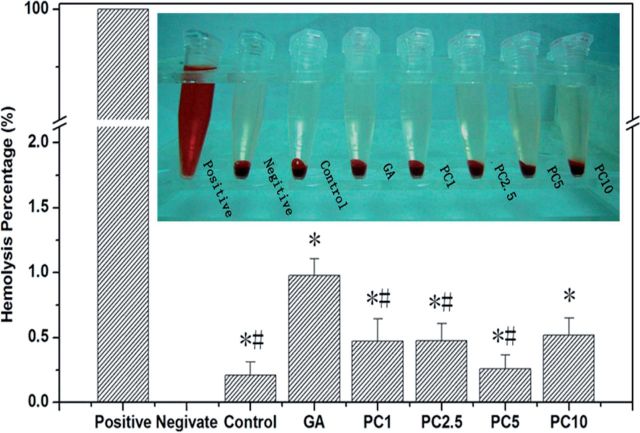
The hemolysis and hemolytic rate of the PC-crosslinked decellularized bovine pericardium ECM. (Reprinted with permission from Ma Bing *et al.* Journal of East China Normal University 2013;**5**:61–79.)

PC can crosslink the decellularized ECM to produce soft matrixes and the stability, mechanical properties and *in vitro* enzyme degradation resistance are significantly enhanced (Fig. 4C and D). In addition, PC has no inhibition for the cell proliferation (Fig. 5), and PC-crosslinked scaffold materials have good biocompatibility (Fig. 6) and hemolysis (Fig. 7) [52, 62].

### Anti-calcification effect of PC

**Figure 8.**
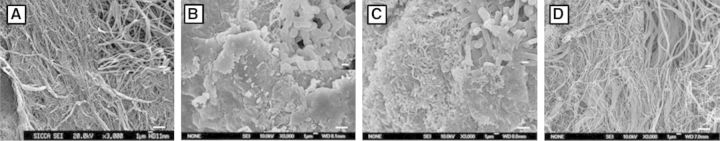
Mineralization of decellularized porcine aortic valves soaked in SBF. (**A**) Decellularized valve matrix before soaking, (**B**) decellularized valves after soaking for 20 days. (**C**) Glutaraldehyde-crosslinked decellularized valves after soaking for 20 days. (**D**) PC-crosslinked decellularized valves after soaking for 20 days. (Reprinted with permission from Ref. [52].)

Calcification in human valves occurs commonly, and is enhanced in damaged valves with congenital anomalies or rheumatic valvulitis [65]. The calcification mechanism is incompletely understood and some hypothesis theories have been proposed. One of the main roles that have been implicated in calcification is organic matrix compositions, including collagen, elastin and other noncollagenous proteins [66–68]. In recent years, researchers have found that elastin, an important component of cardiovascular prostheses, is one of the main causes of calcification, which plays a critical role in the long-term implantation of the prostheses [69]. Calcification that occurs in arteries includes intimal calcification mainly associated with cells and collagen and medial calcification associated with elastin [69, 70]. Although GA could adequately crosslink the collagen component to resist collagenase, it is unable to protect elastin against enzymatic degeneration [50], and the degeneration of elastin may lead to the loss of elastic recoil and calcification [30]. PC can specifically bind to the hydrophobic regions in proline-rich collagen and elastin, form multiple hydrogen bonds and effectively protect elastin from enzyme degradation and inhibit the elastin-associated calcification (Fig. 8) [71].

**Figure 9.**
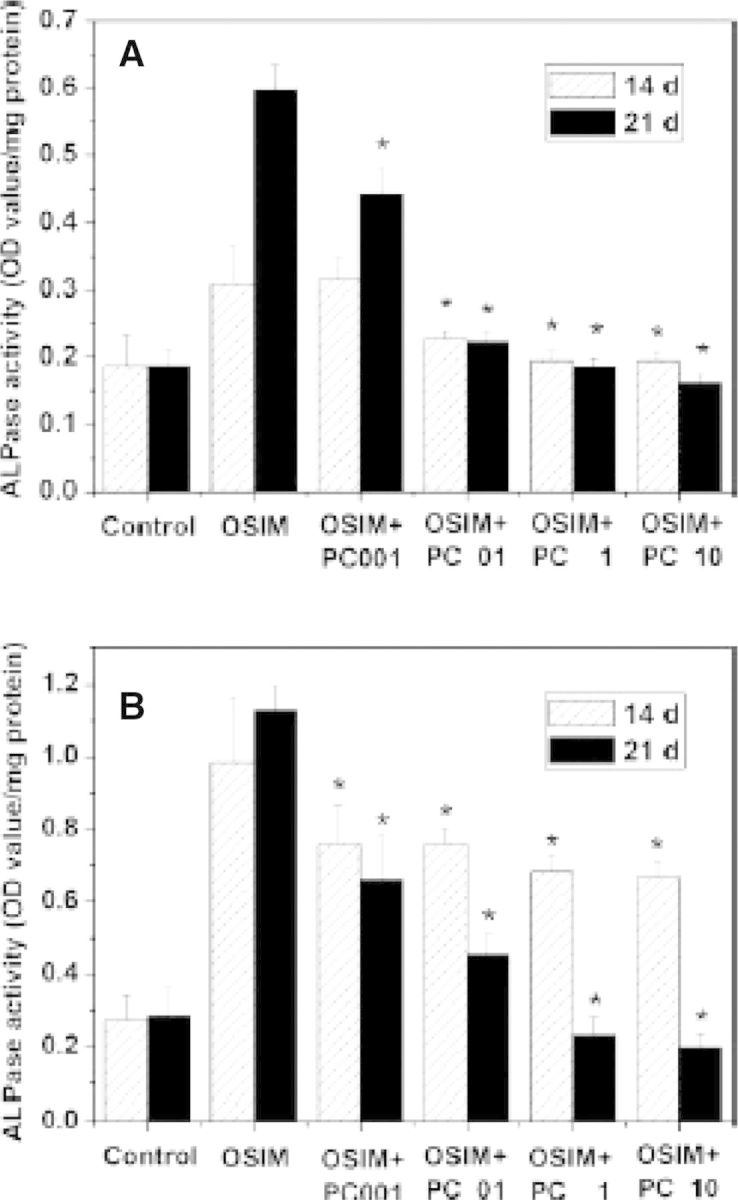
Inhibition effect of PC on ALPase activity of valvular-related cells (n 5 4). (**A**) VICs; (**B**) MSCs. OSIM, osteosynthesis inducing medium; PC, procyanidins. PC001, PC01, PC1 and PC10 represent 0.01, 0.1, 1 and 10 µg/ml PC, respectively. *P < 0.01 compared with OSIM group. (Reprinted with permission from Ref. [52].)

**Figure 10.**
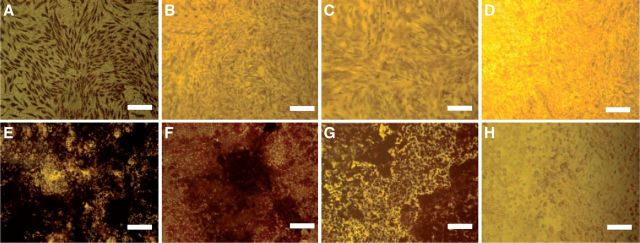
The von Kossa staining of VICs (**A–D**) and MSCs (**E–H**) after osteoinduction (*n* = 4). (A) OSIM; (B) OSIM + 1 µg/ml PC; (C) OSIM + 10 µg/ml PC; (D) control; (E) OSIM; (F) OSIM + 1 µg/ml PC; (G) OSIM + 10 µg/ml PC; (H) control. OSIM, osteosynthesis-inducing medium. Bars = 150 µm. (Reprinted with permission from Ref. [52].)

Cell injury also plays an important role in calcification, which may increase Ca^2+^ and cytosolic phosphate in cells [72]. Studies have shown that bone-marrow-derived mesenchymal stem cells used in tissue engineering and cardiovascular-derived cells, such as valvular interstitial cells (VICs) and vascular smooth muscle cells (VSMCs), could differentiate into osteoblast-like cells which express bone formation biomarkers such as alkaline phosphatase (ALPase) and osteocalcine [73, 74]. In our study, we have proved that PC could inhibit ALPase (Fig. 9) activity and mineral deposition (Fig. 10) of VICs and MSCs. It also can decrease the content of Ca^2+^ and ${\text{PO}}_{\text{4}}^{\text{3}-}$ in cells. It is shown that PC-crosslinked decellularized materials can block calcium phosphate nucleation and suppress mineral deposition, and effectively inhibit calcification [62].

## Conclusions and Future Perspective

In this review, we summarized the research progress of ECM-derived scaffolds and crosslinking strategies for preparation of cardiovascular scaffolds. ECM retains intact organized structure in favors of cell adhesion and proliferation, and they are the promising materials for fabricating cardiovascular scaffolds. Moreover, crosslinking is one of the most important strategies to improve the durability of ECM-derived scaffolds, prevent the degeneration of the materials and enhance the mechanical strength of the scaffolds. The most commonly studied crosslinking agents including chemical crosslinking agents and natural crosslinking agents have been reviewed. The chemical crosslinking agents, such as GA, EDC and pEPC, may have toxicity and result in immune response and calcification. Natural crosslinking agents, such as GP, NDGA, TA and PC, are obtained from plants and have no toxic effect on the human body. Furthermore, PC has the ability to completely inhibit calcification *in vitro* and *in vivo*, and shown promising potential for crosslinking the ECM-derived scaffolds due to its good biocompatibility and anti-calcification activity. Future studies should focus on the optimization of the PC crosslinking method, and detailed evaluation of the performance of PC-crosslinked ECM-derived scaffolds, such as the mechanical strength, long-term durability, degradation rate and biocompatibility, which need future experimental verification *in vitro* and *in vivo*, and further explore the mechanisms of crosslinking and anti-calcification effect of PC.
